# Diagnostic accuracy of CT-based radiomics for predicting occult lymph node metastasis in early-stage lung adenocarcinoma: a systematic review and meta-analysis

**DOI:** 10.3389/fmed.2026.1802716

**Published:** 2026-04-02

**Authors:** Jiarong Huang, Ya Su, Lu Chen, Yuqin Long

**Affiliations:** 1 Department of Respiratory and Critical Care Medicine, The Affiliated Dazu’s Hospital of Chongqing Medical University, Chongqing, China; 2 Department of Medical Affairs, The Affiliated Dazu’s Hospital of Chongqing Medical University, Chongqing, China

**Keywords:** diagnostic accuracy, lung adenocarcinoma, meta-analysis, occult lymph node metastasis, radiomics

## Abstract

**Background:**

This study aims to evaluate the diagnostic accuracy of CT-based radiomics for predicting occult lymph node metastasis (OLNM) in early-stage lung adenocarcinoma.

**Methods:**

Relevant studies up to December 2025 were systematically searched in the databases of PubMed, Embase, Cochrane Library, and Web of Science. Diagnostic accuracy was assessed by pooled estimates of sensitivity, specificity, likelihood ratios, the diagnostic odds ratio, and the summary receiver operating characteristic curve. Subgroup analyses was then conducted to determine sources of heterogeneity.

**Results:**

This study evaluated 10 articles and 6,349 patients. By meta-analysis, CT-based radiomics demonstrated good diagnostic performance for OLNM. The pooled sensitivity and specificity were 0.85 and 0.78 in internal validation cohorts and 0.72 and 0.75 in external validation cohorts, respectively. The area under the summary receiver operating characteristic curve was 0.89 and 0.80 for internal and external validation, respectively. Subgroup analyses of the external validation cohorts suggested possible variation in diagnostic performance according to sample size, CT protocol, and segmentation method.

**Conclusion:**

CT-based radiomics shows potential for non-invasive prediction of OLNM in early-stage lung adenocarcinoma. Further multicenter prospective studies with harmonized imaging and radiomics pipelines are needed to confirm its clinical applicability.

**Systematic review registration:**

CRD420261299869.

## Introduction

Lung cancer remains one of the leading causes of cancer-related morbidity and mortality worldwide and poses a major public health burden (1). Non-small cell lung cancer (NSCLC) occupies about 85% of the total incidence of lung cancer cases, among which lung adenocarcinoma is the most common type (2). Lymph node metastasis (LNM) is common in NSCLC, with an overall prevalence of approximately 40%. Among these patients, N1-stage is observed in 13.9% of individuals along with N1 and N2 metastasis in 16.7% of people, whereas 9.0% of individuals demonstrate isolated N2 metastasis (3, 4). The presence of nodal metastasis affects the prognosis as well as treatment plans for these individuals in NSCLC. This issue is also relevant in early-stage lung adenocarcinoma, in which some patients are classified as clinical N0 before surgery but are subsequently found to have occult lymph node metastasis (OLNM), defined as “LNM not apparent by presurgical imaging” is observed post-operatively by pathology results (5, 6).

Preoperative lymph node evaluation for lung adenocarcinoma is mainly performed through procedures like endobronchial ultrasound-guided transbronchial needle aspiration (EBUS-TBNA), which is considered the standard approach for the evaluation of lymph nodes. However, invasiveness of the technique might restrict its use (7). The mainstay of non-invasive methods, therefore, lies with imaging techniques, and conventional imaging modalities such as Computed Tomography (CT) scans and integrated positron emission tomography (PET)/CT scans are currently used for assessing lymph node involvement. Although 18F-FDG PET/CT generally outperforms CT alone for nodal staging, its detection of microscopic or occult metastasis remains limited by spatial resolution and partial volume effects, and its higher cost may limit routine use (8, 9). Meanwhile, even though CT scans are currently in extensive use for the evaluation of diagnoses as well as assessment for staging of lung cancer, the sensitivity and specificity for early LNM, especially for the assessment of micrometastases, which are frequently encountered in the early stages of lung adenocarcinoma, are considered to be suboptimal.

Aided by the ability to derive quantitative information from imaging modalities, radiomics has significant potential as a diagnostic tool for cancer, including lung cancer, as it helps to determine predicted aspects of tumors as well as metastasis (10, 11). In particular, CT-based radiomics has been utilized to predict LNM. This is a significant aspect of cancer staging and a determinant of patient survival (12). Previous studies have concluded that CT-based radiomics is more effective than traditional approaches for predicting LNM in NSCLC. This is particularly useful in situations where traditional methods of evaluation have failed (13). There has been more recent work that validates its capacity to distinguish between benign and malignancy for lymph nodes and predictive of occult lymph node metastasis in early-stage lung adenocarcinoma (14).

Though there are promising findings for radiomics, there are limited studies focusing specifically on OLNM prediction by radiomics on CT images for lung adenocarcinoma patients. In this regard, there has never been a comprehensive assessment to evaluate the performance of OLNM prediction by radiomics based on CT images for lung adenocarcinoma patients. The aim of this study is to assess the diagnostic performance of radiomics models based on CT images for OLNM prediction in patients with early-stage lung adenocarcinoma.

## Methods

This systematic review was conducted by following the process outlined in the Cochrane Handbook for Systematic Reviews and reported in a way that adheres to the Preferred Reporting Items for Systematic Reviews and Meta-Analyses (PRISMA) statements. Ethical approval was not required due to the nature of systematic review. It has been registered on the international prospective register of systematic reviews website (registration ID: CRD420261299869).

### Search strategy

We carried out a wide-ranging search through various databases, including PubMed, Embase, the Cochrane Library, and Web of Science, with a wide range of search terms, for example, “lung adenocarcinoma,” “non-small cell lung cancer,” “NSCLC,” “lymph node metastasis,” “occult lymph node metastasis,” “radiomics,” “CT radiomics,” “machine learning,” “deep learning,” “texture analysis,” “early-stage,” “Stage I,” “cT1,” and “cT2.” In order to ensure a degree of replicability and ease of later evaluation, the search strategies carried out for each database are set out in full in Supplementary Table S1. In addition, we screened the reference lists of existing systematic reviews/meta-analyses related to our study for further relevant studies.

### Study selection

The inclusion criteria were as follows: (1) original studies including patients with preoperative clinical stages I−II (T1−2N0M0) lung adenocarcinoma confirmed by pathology; (2) studies included patients with preoperative imaging showing no evidence of LNM, and postoperative pathology confirmed the presence or absence of LNM; (3) studies that evaluate the diagnostic accuracy of CT-based radiomics for predicting LNM; (4) studies that present sufficient data to calculate true positive (TP), false positive (FP), true negative (TN), and false negative (FN); (5) full-text articles published in English. The exclusion criteria were as follows: (1) conference abstracts, case reports, letters, editorials, or review articles; (2) studies that included patients with non-early-stage lung adenocarcinoma (Stage III/IV); (3) studies that included patients with preoperative imaging confirming LNM; (4) studies that did not use CT-based radiomics analysis or did not report relevant imaging features; (5) studies that did not provide or could not provide sufficient diagnostic accuracy metrics.

### Data extraction

We used a standardized table for guiding the extraction of the data. Two independent reviewers extracted the following variables for each selected study: the first author, year of publication, country, duration of the study, study design, size of the study, demographics (such as participant age and gender), lymph node staging criteria, clinical stage, the mean size of the tumor, density of the tumor, CT protocol, type of validation set, method of segmentation, region of interest (ROI), image preprocessing, method of radiomics feature extraction, features extracted, feature selection, method of radiomics modeling, and outcomes related to diagnostic accuracy. In cases in which more than one predictive model existed in a study, the model with the greatest AUC or C statistics was selected.

### Quality assessment

Two investigators assessed the methodological quality of included studies by using the Radiomics Quality Score (RQS), including 16 items with a total score of 36 (15). Two reviewers independently rated the quality of all eligible studies for their methodological quality with a standardized approach, with concordance or adjudication by a third party in the event of any discrepancy. The quality assessment tool used was the Quality Assessment of Diagnostic Accuracy Studies (QUADAS)-2, which consists of two core realms: risk of bias and concerns about applicability (16).

### Statistical analysis

All analysis of results was carried out using Stata software, version 18.0. The level of heterogeneity among study results was measured using I^2^ statistics, which was low (<25%), moderate (25–50%), or substantial (>50%) when >5% of overall variability was not explained. If there were statistically significant heterogeneity, results would be pooled using a random effects model. Otherwise, results would be pooled using a fixed effects model. Pooled estimates of sensitivity, specificity, positive likelihood ratio (PLR), negative likelihood ratio (NLR), and diagnostic odds ratio (DOR) would be calculated along with their respective 95% confidence intervals. Summary receiver operating characteristic (SROC) plots would be created to provide an overall summary of diagnostic accuracy. Sub-group analysis would be carried out according to sample size, tumor diameter, clinical stage, CT protocol, and segmentation method to explore sources of heterogeneity. To examine the robustness of the pooled estimates, a leave-one-out sensitivity analysis was performed. Publication bias was assessed using Deek’s funnel plot asymmetry test. Statistical significance was set at *p* < 0.05.

## Results

### Literature search

**Figure 1:**
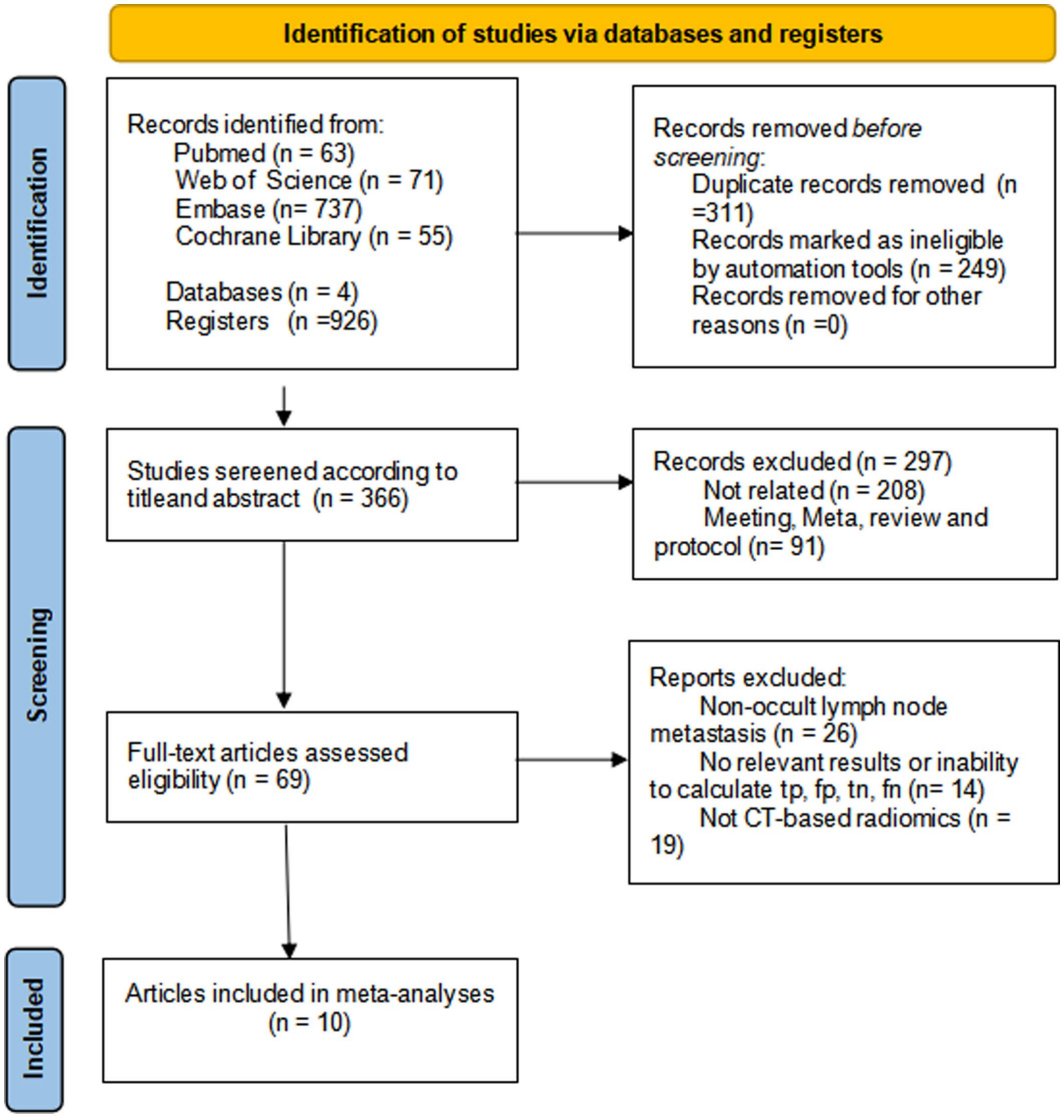
Flow diagram of study selection.

Systematic literature searching on PubMed, Web of Science, Cochrane Library, and Embase resulted in 926 records. Ineligible records and duplicates were automatically filtered through programming, and 366 studies were left to be included. Title and abstract screening led to the exclusion of 297 papers, leaving 69 for full-text review. After assessing these, 10 studies were included in the meta-analysis (14, 17–25) (Figure 1).

### Characteristics of included studies

The studies included in the analysis were all conducted in China. The sample sizes ranged from 216 to 1,325 participants, totaling 6,349 individuals, mostly female (*n* = 3,467). The average age of participants ranged from 57.2 to 61.4 years. The included studies all employed a retrospective design, with 2 studies being single-center and 8 studies being multi-center. All studies relied on surgical resection for the diagnosis of LNM. The LNM staging was based on either the American Joint Committee on Cancer (AJCC) 8th edition or the International Association for the Study of Lung Cancer (IASLC) 8th edition, and all participants had clinical stages I–II lung adenocarcinoma. The clinical stages varied, with 3 studies including patients with cT1–2N0M0, 5 with cT1N0M0, and 1 each with cT1a-bN0M0 and cT1 − 2bN0M0. Tumor diameters ranged from 1.7 cm to 2.5 cm, and tumor densities varied, including solid, part-solid, and ground glass opacity (GGO) in some studies. In terms of CT types, 5 studies used enhanced CT, 2 studies used unenhanced CT, and 3 studies employed both enhanced and unenhanced CT imaging. Six studies utilized both internal and external validation sets, while 2 studies used only internal validation and 2 studies used only external validation (Table 1 and Supplementary Table S2).

**Table 1:** Characteristics of included studies.

ID	Country	Study period	Study design	No. of patients	Age	Gender (M/F)	Lymph node staging criteria	Clinical stage	Tumor diameter	Tumor density	CT protocol	Validation
Zhong et al. (2018) (17)	China	2013–2016	Retrospective, single center	492	Mean, 61.4 ± 9.7	173/319	AJCC 8th	cT1–2N0M0	Mean, 2.0 ± 1.5 cm	−	Unenhanced CT	Internal
Das et al. (2021) (18)	China	2016–2019	Retrospective, multicenter	216	Mean, 61.2 ± 14.9	128/88	AJCC 8th	cT1N0M0	Mean, 2.3 ± 0.6 cm	Solid, part-solid, GGO	Enhanced CT	Internal and external
Zhang et al. (2021) (19)	China	2014–2018	Retrospective, multicenter	244	Mean, 61.3 ± 1.3	106/138	AJCC 8th	cT1-2N0M0	Mean, 2.5 ± 0.4 cm	Solid	Enhanced CT	External
Liu et al. (2024) (20)	China	2005–2017	Retrospective, single center	258	Median, 59.0 (51.0, 66.0)	116/142	IASLC 8th	cT1N0M0	Median, 1.9 (1.5, 2.5) cm	Solid, part-solid, GGO	Enhanced CT	Internal
Tian et al. (2024) (21)	China	2011–2022	Retrospective, multicenter	1,325	Mean, 60.2 ± 0.7	−	−	cT1a-bN0M0	Mean, 1.7 ± 0.1 cm	Solid, part-solid	Unenhanced and enhanced CT	Internal and external
Ye et al. (2024) (22)	China	2020–2022	Retrospective, multicenter	473	Mean, 59.2 ± 9.6	205/268	IASLC 8th	cT1N0M0	Mean, 1.8 ± 0.5 cm	Solid, part-solid	Unenhanced CT	External
Huang et al. (2025) (14)	China	2016–2023	Retrospective, multicenter	1,132	Mean, 57.5 ± 9.8	458/675	IASLC 8th	cT1N0M0	Mean, 1.7 ± 0.6 cm	Solid, part-solid, GGO	Enhanced CT	Internal and external
Huang et al. (2025) (23)	China	2016–2023	Retrospective, multicenter	1,099	Mean, 57.2 ± 10.1	441/658	IASLC 8th	cT1N0M0	Mean, 1.7 ± 0.6 cm	Solid, part-solid, GGO	Enhanced CT	Internal and external
Yin et al. (2025) (24)	China	2018–2021	Retrospective, multicenter	358	Median, 59.7 (54.8, 67.3)	175/183	AJCC 8th	cT1−2bN0M0	Median, 2.4 (1.9, 3.1) cm	Solid, part-solid, GGO	Unenhanced CT	Internal and external
Zhao et al. (2025) (25)	China	2016–2024	Retrospective, multicenter	752	Mean, 59.4 ± 8.5	298/454	AJCC 8th	cT1−2N0M0	Mean, 2.0 ± 0.7 cm	Solid, part-solid, GGO	Enhanced CT	Internal and external

M, male; F, female; LNM, lymph node metastasis; CT, computerized tomography; IASLC, the International Association for the Study of Lung Cancer; GGO, ground glass opacity; AJCC, American Joint Committee on Cancer.

### Radiomics feature extraction and modeling approaches

Table 2 presents the radiomics workflow. The segmentation method was primarily manual (8 studies), with semi-automatic and automatic segmentation methods used in 1 study each. All studies focused on the primary tumor-only region as the ROI. Common image preprocessing methods included z-score normalization (4 studies), HU values normalization (4 studies), and voxel spacing resampling (3 studies). The PyRadiomics tool was used for radiomics extraction in 7 studies, while MaZda and Artificial Intelligence Kit were used in some studies. Feature selection methods were predominantly based on Least Absolute Shrinkage and Selection Operator (LASSO) in 8 studies, followed by Principal Component Analysis (PCA) in 3 studies, Minimum Redundancy Maximum Relevance (MRMR) in 1 study, and Relief in 1 study. For modeling approaches, machine learning methods such as support vector machine (SVM), XGBoost, and logistic regression (LR) were used in 8 studies, while deep learning approaches such as ResNet and DLRad were employed in 6 studies, and traditional statistics in 2 studies.

**Table 2:** Radiomics feature extraction and modeling approaches.

ID	Segmentation method	ROI	Image preprocessing	Radiomics extraction method	Radiomics feature	Feature selection	Radiomics modeling approach
Zhong et al. (2018) (17)	Manual, Analysis Kit 3.0.0	Primary tumor-only	Adjust pixel values to zero mean and unit variance	MaZda	300 total (first-order, texture)	Reproducibility and Redundancy Filtering; Relief; PCA (4 features)	Machine learning (SVM)
Das et al. (2021) (18)	Manual, ITK-SNAP 3.6.0	GTV, PTV, LN	Voxel interval adjustment, denoising, intensity discretization	Artificial Intelligence Kit	290 total (shape, first-order, texture)	LASSO (12 features)	Traditional statistics (LR)
Zhang et al. (2021) (19)	Manual	Primary tumor-only	−	PyRadiomics	490 total (shape, first-order, texture)	LASSO, multivariable logistic regression (3 features)	LASSO
Liu et al. (2024) (20)	Manual, ITK-SNAP	Primary tumor-only	Linear interpolation and grayscale discretization	Artificial Intelligence Kit	107 total (shape, first-order, texture)	MRMR, LASSO (6 features)	Machine learning (XGBoost)
Tian et al. (2024) (21)	Automatic, uAI Research Portal	Primary tumor-only	Bilinear and nearest-neighbor interpolation	uAI Research Portal	1,364 total (shape, first-order, texture)	Decision tree (10 features)	Deep learning (ResNet-18)
Ye et al. (2024) (22)	Manual, ITK-SNAP 3.8.0	Primary tumor-only	*z*-score normalization	PyRadiomics	268 total (shape, first-order, texture)	LASSO (11 features)	DTL (ResNet50 + LR)
Huang et al. (2025) (14)	Manual, ITK-SNAP 4.2.0	Primary tumor-only	HU values normalization; voxel spacing resampling	PyRadiomics	1834 total (shape, first-order, texture)	LASSO (28 features)	Machine learning (XGBoost)
Huang et al. (2025) (23)	Manual, ITK-SNAP 4.2.0	Primary tumor-only	HU values normalization; voxel spacing resampling	PyRadiomics	1834 total (shape, first-order, texture)	Univariate and multivariable logistic regression (7 features)	Deep learning (DLRad)
Yin et al. (2025) (24)	Manual, ITK-SNAP 3.8.0	Primary tumor-only	*z*-score normalization	PyRadiomics	1946 total (shape, first-order, texture)	Univariate and multivariable logistic regression (7 features)	Deep learning (3D SE-ResNet34)
Zhao et al. (2025) (25)	Semi-automatic, 3D-Slicer v5.8.1	Primary tumor-only	HU values normalization; voxel spacing resampling	PyRadiomics	1,316 total (shape, first-order, texture)	PCA, LASSO (7 features)	Machine learning (XGBoost)

ROI, region of interest; PCA, principal component analysis; SVM, support vector machine; GTV, gross tumor volume; PTV, peritumoral volume; LASSO, least absolute shrinkage and selection operator; DTL, deep transfer learning; LR, logistic regression; MRMR, minimum redundancy maximum relevance.

### Quality assessment

**Figure 2:**
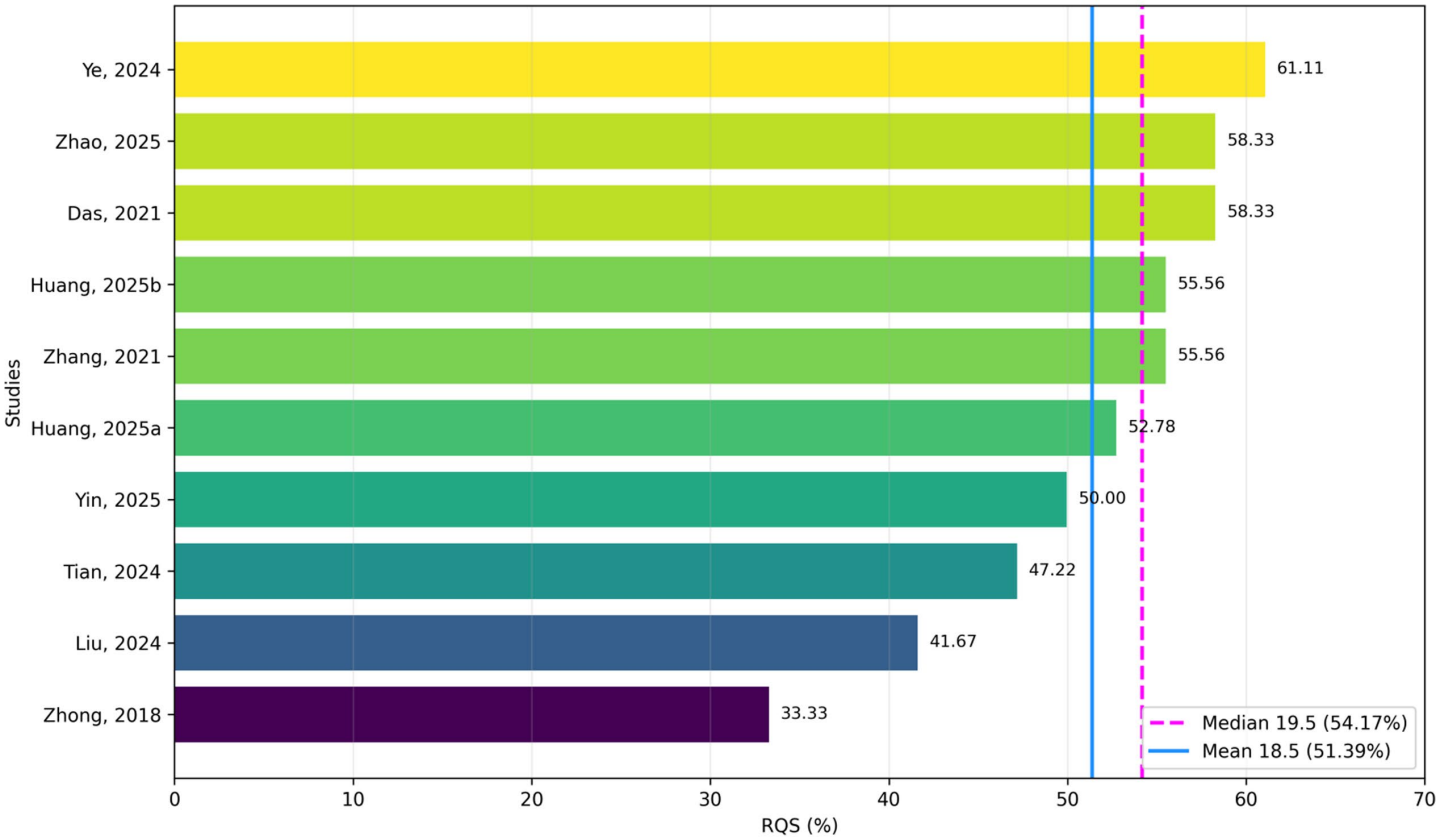
Distribution of radiomics quality score percentages across the included studies.

**Figure 3:**
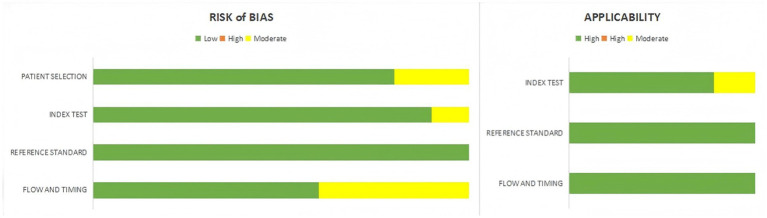
Quality assessments of included studies using the QUADAS-2 tool.

As shown in Supplementary Table S3 and Figure 2, the RQS of the included studies ranged from 12 to 22 (corresponding to 33.33–61.11% of the maximum score), with a median RQS of 19.5 (17, 21). The quality assessment of the included studies using the QUADAS-2 tool generally had variable overall quality. Overall, the quality of the 10 studies is good, with most rated as low risk for reference standard and index test, indicating strong methodology. However, several studies were rated as unclear in risk of bias and applicability, particularly in patient selection, index test, and flow and timing. Specifically, patient selection was unclear in 2 studies due to retrospective bias; index test was unclear in 1 study due to multi-center data inconsistencies; and flow and timing were unclear in 4 studies because of incomplete reporting of the diagnostic workflow or follow-up information. In applicability, patient selection was unclear in 2 studies due to sample limitations (Figure 3).

### Diagnostic performance

**Figure 4:**
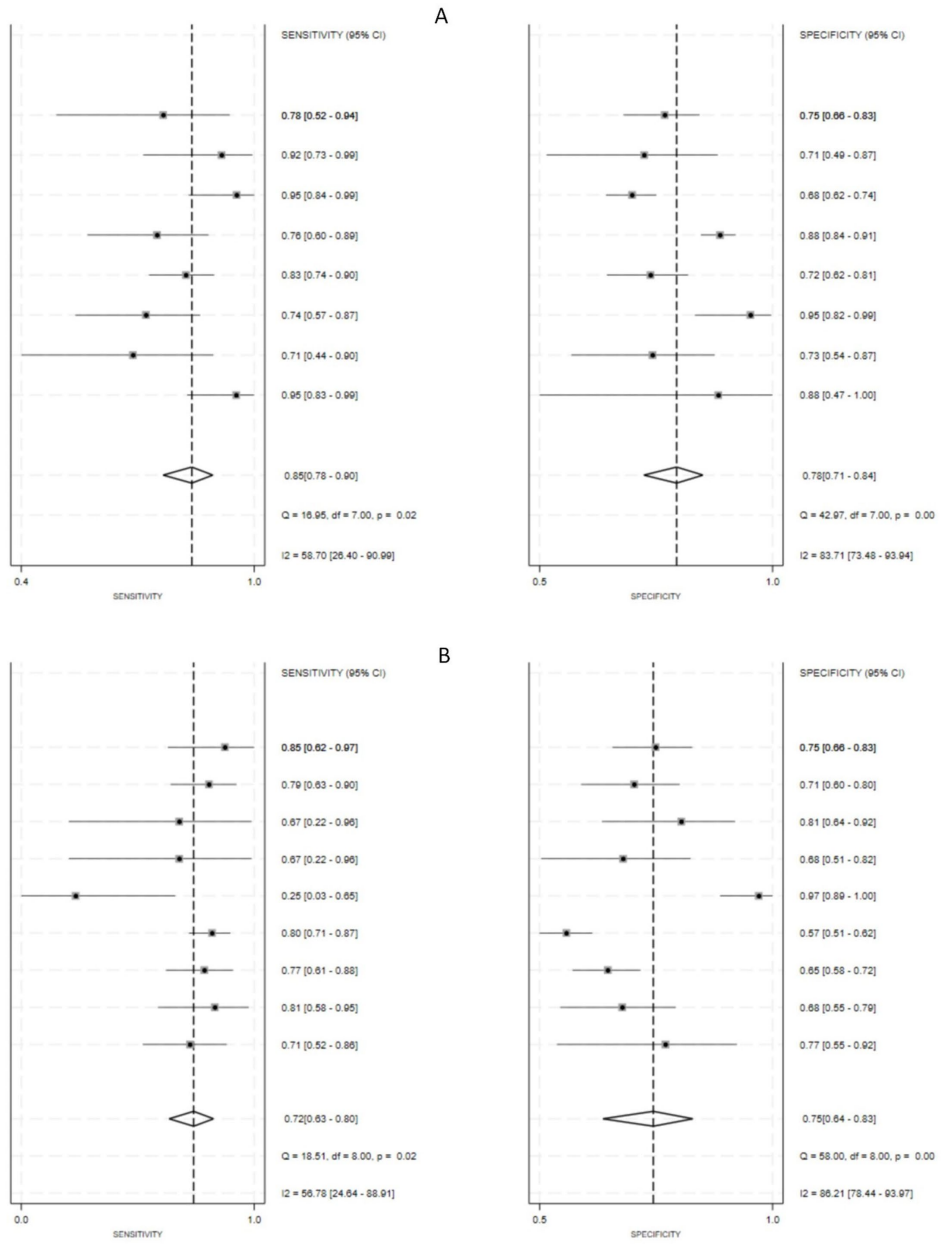
Forest plots of sensitivity and specificity with corresponding heterogeneity statistics: **(A)** internal validation cohorts; **(B)** external validation.

**Figure 5:**
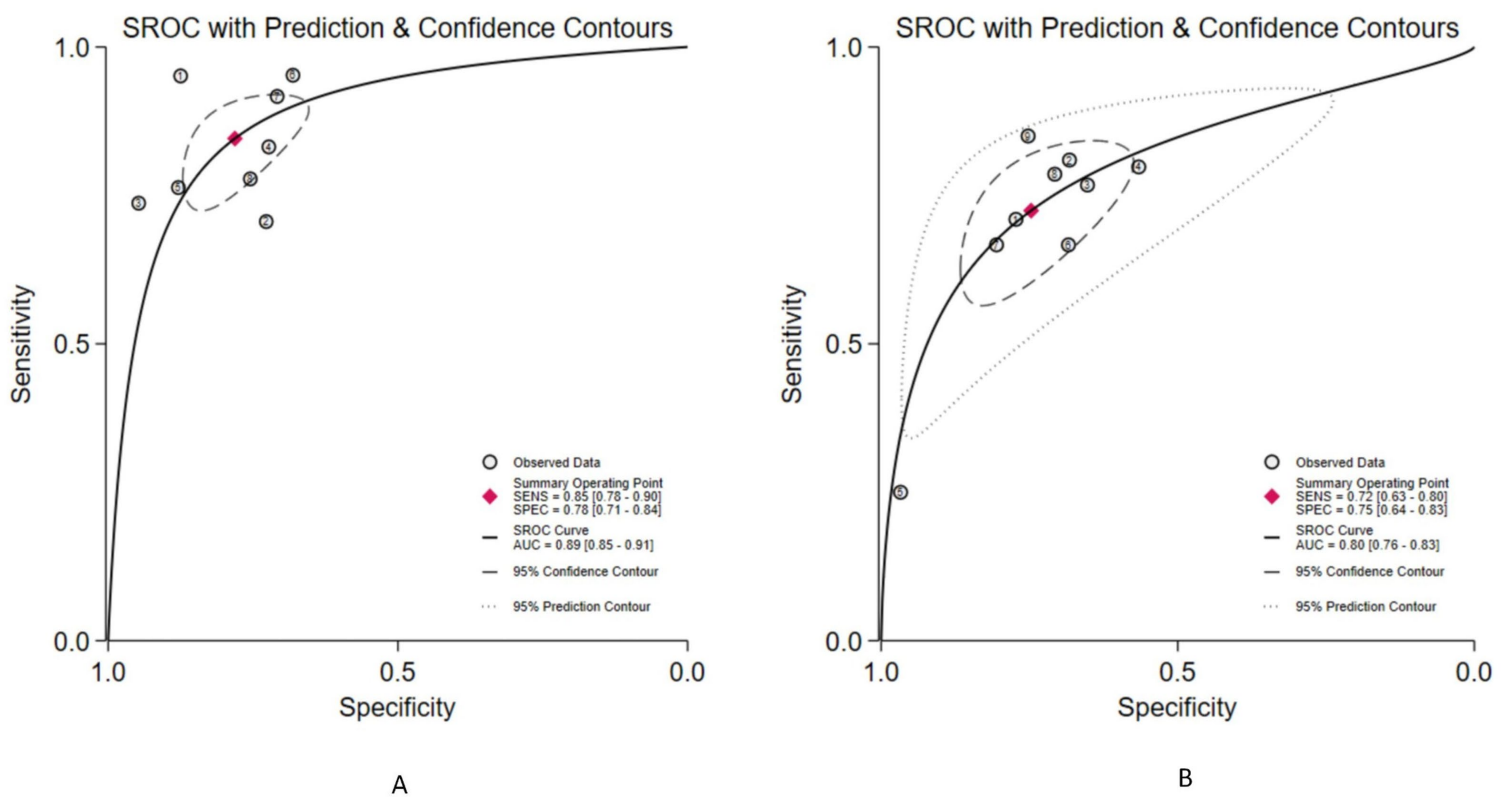
Summary receiver operating characteristic curve for the diagnostic accuracy of CT-based radiomics in predicting occult lymph node metastasis (**A**, internal validation cohorts; **B**, external validation).

Eight studies evaluated the diagnostic performance using an internal validation set (14, 17, 18, 20, 21, 23–25), while nine cohorts from eight studies assessed the diagnostic performance on external validation sets (14, 18, 19, 21–25). In internal validation set, the pooled sensitivity for the detection of OLNM by CT-based radiomics was 0.85 (95% CI, 0.78–0.90), and the pooled specificity was 0.78 (95% CI, 0.71–0.84). These were accompanied by corresponding *I*^2^ values of 58.70 and 83.71%, respectively, which implies substantial heterogeneity across the included studies (Figure 4A). The pooled PLR was 3.87 (95% CI, 2.95–5.06), the pooled NLR was 0.20 (95% CI, 0.14–0.28), and its corresponding DOR was 19.60 (95% CI, 13.37–28.72) (Supplementary Figures S1A, S2A). The SROC curve revealed an excellent overall diagnostic performance, with an AUC of 0.89 (95% CI, 0.85–0.91) (Figure 5A). In external validation set, the pooled sensitivity for the detection of OLNM by CT-based radiomics was 0.72 (95% CI, 0.63–0.80), and the pooled specificity was 0.75 (95% CI, 0.64–0.83). These were accompanied by corresponding I^2^ values of 56.78 and 86.21%, respectively, which implies substantial heterogeneity across the included studies (Figure 4B). The pooled PLR was 2.87 (95% CI, 2.10–3.91), the pooled NLR was 0.37 (95% CI, 0.28–0.48), and its corresponding DOR was 7.77 (95% CI, 5.15–11.70) (Supplementary Figures S1B, S2B). The SROC curve revealed a good overall diagnostic performance, with an AUC of 0.80 (95% CI, 0.76–0.83) (Figure 5B).

### Subgroup analysis

We performed subgroup analyses based on sample size, tumor diameter, clinical stage, CT protocol, and segmentation method in external validation cohorts to explore potential sources of heterogeneity (Table 3). Studies with a sample size < 1,000 demonstrated a pooled sensitivity of 0.71 (95% CI, 0.50–0.85) and specificity of 0.81 (95% CI, 0.66–0.90), with an AUC of 0.83 (95% CI, 0.79–0.86), whereas those with ≥1,000 patients yielded a sensitivity of 0.76 (95% CI, 0.64–0.80), specificity of 0.66 (95% CI, 0.56–0.74), and an AUC of 0.78 (95% CI, 0.74–0.81). No significant between-group difference was observed in sensitivity (*p* = 0.61), whereas the difference in AUC was significant (*p* < 0.05). For studies with a reported mean tumor diameter <2 cm and ≥2 cm, the pooled sensitivity and specificity were 0.65 and 0.77 with an AUC of 0.77, and 0.78 and 0.73 with an AUC of 0.82, respectively; no significant between-group difference was observed in sensitivity (*p* = 0.22), whereas the difference in AUC was of borderline significance (*p* < 0.05). Enhanced CT–based radiomics showed a higher AUC (0.81; 95% CI, 0.78–0.85) than unenhanced CT (AUC, 0.73; 95% CI, 0.68–0.78), while manually segmented models achieved an AUC of 0.81 (95% CI, 0.77–0.84). There was no significant between-group difference in sensitivity (*p* = 0.86), whereas the AUC was significantly higher in the enhanced CT subgroup (*p* = 0.01). These results suggest that differences in sample size, tumor diameter, CT protocol, and segmentation method may represent potential sources of the observed heterogeneity across studies.

**Table 3:** Results of subgroup analyses for diagnostic accuracy in external validation cohorts.

Subgroup	No. of studies	Sensitivity (95% CI)	Specificity (95% CI)	PLR (95% CI)	NLR (95% CI)	DOR (95% CI)	AUC (95% CI)
Sample size							
<1,000	5	0.71 (0.50–0.85)	0.81 (0.66–0.90)	3.63 (2.30–5.73)	0.36 (0.22–0.60)	9.97 (5.65–17.59)	0.83 (0.79–0.86)
≥1,000	4	0.76 (0.64–0.80)	0.66 (0.56–0.74)	2.20 (1.72–2.81)	0.36 (0.21–0.62)	5.93 (3.42–10.29)	0.78 (0.74–0.81)
*p-*value		*p* = 0.61	*p* = 0.04	*p* = 0.12	*p* = 0.99	*p* = 0.25	*p* = 0.04
Mean tumor diameter							
<2 cm	5	0.65 (0.43–0.82)	0.77 (0.57–0.89)	2.83 (1.71–4.69)	0.46 (0.30–0.70)	6.20 (3.37–11.39)	0.77 (0.73–0.80)
≥2 cm	4	0.78 (0.70–0.85)	0.73 (0.67–0.77)	2.84 (2.29–3.52)	0.30 (0.21–0.43)	9.39 (5.61–15.71)	0.82 (0.78–0.85)
*p-*value		*p* = 0.22	*p* = 0.64	*p* = 0.96	*p* = 0.17	*p* = 0.33	*p* = 0.04
Clinical stage							
cT1	6	0.75 (0.68–0.81)	0.65 (0.62–0.69)	2.15 (1.86–2.49)	0.39 (0.30–0.50)	5.54 (3.69–8.31)	0.74 (0.69–0.79)
cT1-2	3	–	–	–	–	–	–
*p-*value		–	–	–	–	–	–
CT protocol							
Unenhanced	4	0.77 (0.70–0.83)	0.65 (0.61–0.68)	2.16 (1.87–2.49)	0.36 (0.27–0.48)	5.95 (3.83–9.24)	0.73 (0.68–0.78)
Enhanced	5	0.76 (0.66–0.84)	0.74 (0.68–0.78)	2.88 (2.28–3.63)	0.32 (0.22–0.48)	8.89 (5.03–15.71)	0.81 (0.78–0.85)
*p-*value		*p* = 0.86	*p* = 0.01	*p* = 0.06	*p* = 0.64	*p* = 0.33	*p* = 0.01
Segmentation method							
Manual	6	0.68 (0.51–0.82)	0.79 (0.66–0.88)	3.32 (2.22–4.98)	0.40 (0.26–0.61)	8.37 (4.75–14.73)	0.81 (0.77–0.84)
Non-manual	3	–	–	–	–	–	–
*p-*value		–	–	–	–	–	–

PLR, positive likelihood ratio; NLR, negative likelihood ratio; DOR, diagnostic odds ratio; AUC, area under the curve; CI, confidence interval; CT, computed tomography.

### Predictive assessment and likelihood ratios

Based on a pre-test probability of 50% for OLNM, the Fagan plot showed that a positive radiomics result increased the post-test probability to 79% and reduced it to 16% following a negative result in the internal validation cohort (Supplementary Figure S3A), whereas in the external validation cohort, a positive result increased the post-test probability to 74% and a negative result reduced it to 27% (Supplementary Figure S3B). Likelihood ratio scatter plots demonstrated that most studies in the internal validation cohort were located in the right lower quadrant, indicating a moderate confirmatory value of CT-based radiomics models (Supplementary Figure S4A), with a similar but slightly attenuated diagnostic performance observed in the external validation cohort (Supplementary Figure S4B).

### Publication bias and sensitivity analysis

**Figure 6:**
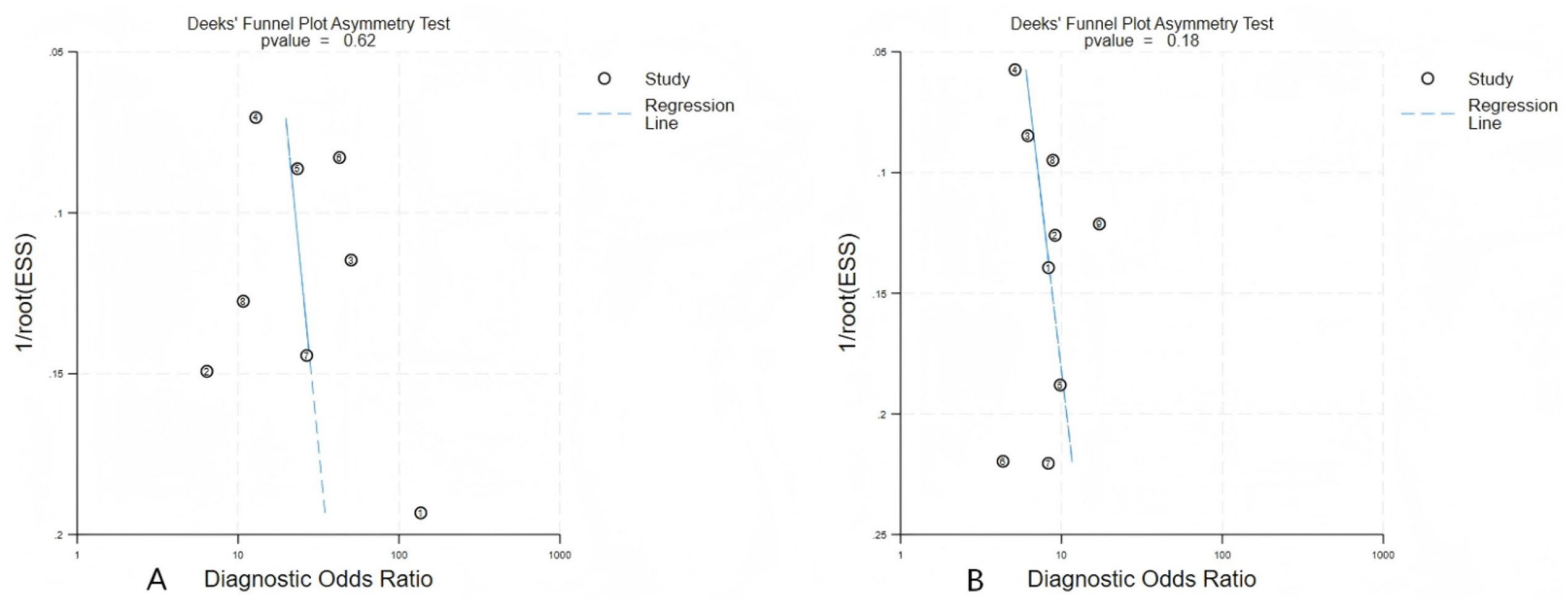
Deek’s funnel plot for assessment of publication bias: **(A)** internal validation cohorts; **(B)** external validation.

Deek’s funnel plot asymmetry test showed no significant evidence of publication bias in either the internal (*p* = 0.62) or external (*p* = 0.18) validation cohorts, indicating a low likelihood of small-study effects (Figures 6A,B). Leave-one-out sensitivity analyses in external validation cohorts showed that the pooled diagnostic performance was not materially altered by the exclusion of any single study, indicating the robustness of the overall results.

## Discussion

This systematic review and meta-analysis evaluates the diagnostic accuracy of CT-based radiomics models for the preoperative assessment of OLNM in patients with early-stage lung adenocarcinoma. The CT-based radiomics approach showed promising discriminative power for OLNM with improved performance for the internal validation set and maintained accuracy for the external validation set. In clinical practice, these results imply that the CT-based radiomics approach may substantially change the probability estimate for OLNM and offer complementary information for the preoperative risk assessment of patients with OLNM. Furthermore, subgroup analyses demonstrated that the extent of the underlying dataset, the used CT scanning protocols, and the methods adopted for the definition of the region of interest influenced the level of the diagnostic accuracy, suggesting the sensitivity of the method might be compromised by the underlying quality and detail of the generated dataset rather than the accuracy of the final clinical assessment per se. Importantly, the high level of the pooled sensitivity for the estimates provided across the analyses indicates the CT-based radiomics approach may be very reliable for detecting patients with OLNM, an issue of specific clinical relevance for the preoperative assessment for the exclusion of patients with potentially missed OLNM lesions among the sentinel lymph nodes or other lymph nodes simultaneously investigated around the primary tumor via preoperative lymphadenopathy techniques and technologies. In support of the interpretability of the estimates provided, sensitivity analyses confirmed the stability of the cumulative estimates, and visual inspections of the combined plot suggested an absence of obvious evidence for publication bias.

Recent years have seen an increasing number of studies that investigate the role of radiomics in the evaluation of LNM in lung cancer. Radiomics allows for the extraction of high-dimensional features of tumor morphology, intensity, and textures from routine imaging, which might represent intratumoral heterogeneity or invasive growth patterns of malignancies that possibly relate to LNM (26–28). Diagnostic accuracy of deep learning and radiomics analysis in lung cancer staging was previously assessed in one study that included six radiomics studies aiming to predict LNM, finding a combined AUC of 0.74 (29). Another meta-analysis investigated the diagnostic accuracy of LNM prediction by using radiomics based on CT or PET/CT imaging representing thoracic LNM in lung adenocarcinoma, finding high accuracy of approximately 0.94; interestingly, this analysis comprised studies that involved LNM within a broad range of disease stages, which comprised different pathological-clinical contexts (30). By contrast, to focus on a specific context representing node-negative, early-stage lung adenocarcinoma, our analysis comprised all available investigations aiming to evaluate OLNM, which might be considered a post facto, but patient-relevant scenario. Although OLNM was reported to have high AUCs (approximately 0.87–0.97) for image-based and radiomics models derived from the primary tumor—exceeding those of clinical or pathological models (AUC, 0.72–0.81)—their review was largely descriptive and most included studies lacked external validation (31). Our meta-analysis was restricted to CT-based radiomics and quantitatively pooled diagnostic performance separately for internal and external validation cohorts, enabling a more robust assessment of model generalizability.

Differences across studies can be attributed to the scale of the study, the size of the tumors, and fundamental aspects of the radiomics pipeline, including CT scan protocols and the methods of segmentation. Sample size can play a significant role in defining the robustness of the diagnostic performance of a study due to the likelihood of overfitting of smaller multicenter samples for feature selection/optimization versus the need for more conservative validation of diagnostic performance in larger multicenter samples that introduce greater variability due to differences in patient mix and scanner differences (32, 33). In our study, one possible explanation for the lower specificity observed in studies with larger sample sizes is the greater heterogeneity of external validation settings, whereas smaller studies may overestimate specificity because of overfitting. Tumor size can be a defining criterion in determining the diagnostic accuracy of radiomics due to higher variability in signal characteristics for larger tumors due to enhanced heterogeneity versus smaller lesions that demonstrate a more subtle signal (34). Variability in CT protocols is a key determinant of inter-study differences that can be due to differences in the use of contrast enhancement and varying differences in tissue thickness or reconstruction algorithms that can significantly influence voxel-wise signal characteristics/intensity and thereby biological information extracted from the study by radiomics (35, 36). Segmentation strategies further contribute to variability by directly defining the regions of interest from which features are extracted. While manual segmentation may provide more precise tumor delineation, it is inherently operator dependent, whereas non-manual approaches can improve efficiency and reproducibility but may be more sensitive to differences in image quality and tumor morphology (37, 38). While these factors suggest that inter-study variability is due to not only clinical factors but differences in the pipeline from images to model development, they emphasize the need for a standardized pipeline that supports robust validation.

To expand on these results, the radiomics model performed better on internal validation than external validation but still maintained a high level of discriminative power when validated externally. The trend of radiomics model performance implies that radiomics has the potential to be utilized for preoperative risk stratification of OLNM for radiologically node-negative, early-stage patients, although the generalizability and application of radiomics models for authentic patient diagnosis and decision-making appear to be model development dataset-dependent (39, 40). The moderate sensitivity and specificity achieved during the external validation of the radiomics model imply that while the radiomics model could assist clinicians in identifying patients at high risk of OLNM, the radiomics model could not be exclusively relied upon to make decisions either for or against the diagnosis of LNM at the patient level (41). Hence, for the time being, radiomics model-based approaches seem less likely to offer direct alternatives to conventional methods of patient staging yet could potentially be utilized as supporting approaches to assist clinicians with the task of patient decision-making and selection of patients for aggressive evaluation of nodal involvement and the development of tailored approaches during surgery (42). The enduring presence of the signal during the external validation stage of the radiomics model implies that radiomics has the ability to extract genuinely important and meaningful information that is not limited to the development dataset, and radiomics could potentially assume important and crucial roles at the patient decision level despite being model development-dataset dependent (43, 44). Future research ought to concentrate on radiomics model validation and the development of potentially consecutive approaches that integrate radiomics parameters with conventional parameters of imaging and patient decision-making.

This work represents a comprehensive assessment of CT-based radiomics for the preoperative prediction of OLNM in early-stage lung adenocarcinoma, with attention to both internal and external validation settings. However, several limitations should be considered. All included studies were retrospective and conducted in Asia, which may limit generalizability to broader populations. In addition, the included studies used different staging systems, including AJCC and IASLC classifications, which may have introduced inconsistency across studies. Substantial methodological heterogeneity existed across studies, particularly in CT acquisition protocols, segmentation strategies, and radiomics pipelines, which may have influenced diagnostic performance. Finally, the limited number of studies with external validation restricted some subgroup analyses and warrants cautious interpretation of the results.

## Conclusion

CT-based radiomics shows promise for preoperative prediction of OLNM in early-stage lung adenocarcinoma, with stronger performance in internal validation and good performance in external validation. Further multicenter prospective validation with harmonized imaging and radiomics pipelines is required to improve generalizability and support clinical translation.

## Data Availability

The original contributions presented in the study are included in the article/Supplementary material, further inquiries can be directed to the corresponding authors.
