# Supplementary material for: Diagnostic accuracy of CT-based radiomics for predicting occult lymph node metastasis in early-stage lung adenocarcinoma: a systematic review and meta-analysis
Source: Front Med (Lausanne). 2026 Apr 2;13:1802716. doi: 10.3389/fmed.2026.1802716 (PMC13083186; doi:10.3389/fmed.2026.1802716)
Supplement: Supplementary file 1 [file Data_Sheet_1.docx]

**Supplementary files**

**List of supplementary files**

Table S1. Database search strategies and results for CT-based radiomics and occult lymph node metastasis.

Table S2. Distribution of lymph node–positive and lymph node–negative cases across training, validation, and external test cohorts in the included studies

Table S3. Radiomic quality scores for all included studies.

Figure S1. Forest plots of PLR and NLR with corresponding heterogeneity statistics. A, internal validation cohorts; B, external validation.

Figure S2. Forest plots of DOR with corresponding heterogeneity statistics. A, internal validation cohorts; B, external validation.

Figure S3. Fagan nomogram of CT-based radiomics for diagnosis of occult lymph node metastasis according to different pretest probabilities (prior). A, internal validation cohorts; B, external validation.

Figure S4. Negative Likelihood ratio scattergram. A, internal validation cohorts; B, external validation.

**Table S1. Database search strategies and results for CT-based radiomics and occult lymph node metastasis.**

| **Database** | **Search Strategy (date: 2025.12.31.)** | **Result** |
| --- | --- | --- |
| PubMed | ("Lung Neoplasms"[MeSH] OR "Lung cancer" OR "Lung adenocarcinoma" OR NSCLC OR "Pulmonary nodule" OR "Ground glass opacity" OR GGO)  AND  ("Lymphatic Metastasis"[MeSH] OR "Lymph node metastasis" OR "Occult metastasis" OR "Nodal involvement" OR "N staging" OR LNM OR N1 OR N2)  AND  ("Radiomics" OR "Deep learning" OR "Machine learning" OR "Artificial intelligence" OR "Texture analysis" OR "Convolutional neural network" OR CNN OR "Nomogram")  AND  ("Stage I" OR "Stage IA" OR "Stage IB" OR "cT1" OR "cT2" OR "Clinical stage I" OR "Early stage" OR "cN0" OR "N0M0") | 63 |
| Embase | ('Lung adenocarcinoma' OR 'NSCLC') AND ('Lymphatic Metastasis'/exp OR 'Occult Lymph node metastasis') AND ('Radiomics' OR 'Deep learning' OR 'Machine learning') AND ('Stage I') | 737 |
| Web of Science | ANYWHERE: ("Lung Neoplasms" OR "Lung cancer" OR "Lung adenocarcinoma" OR "NSCLC" OR "Pulmonary nodule" OR "Ground glass opacity" OR "GGO")  AND  ANYWHERE: ("Lymphatic Metastasis" OR "Lymph node metastasis" OR "Occult metastasis" OR "Nodal involvement" OR "N staging" OR "LNM" OR "N1" OR "N2")  AND  ANYWHERE: ("Radiomics" OR "Deep learning" OR "Machine learning" OR "Artificial intelligence" OR "Texture analysis" OR "Convolutional neural network" OR "CNN" OR "Nomogram")  AND  ANYWHERE: ("Stage I" OR "Stage IA" OR "Stage IB" OR "cT1" OR "cT2" OR "Clinical stage I" OR "Early stage" OR "cN0" OR "N0M0") | 71 |
| Cochrane Library | (("Lung Neoplasms" OR "Lung cancer" OR "Lung adenocarcinoma" OR NSCLC OR "Pulmonary nodule" OR "Ground glass opacity" OR GGO) AND ("Lymphatic Metastasis" OR "Lymph node metastasis" OR "Occult metastasis" OR "Nodal involvement" OR "N staging" OR LNM OR N1 OR N2) AND ("Radiomics" OR "Deep learning" OR "Machine learning" OR "Artificial intelligence" OR "Texture analysis" OR "Convolutional neural network" OR "Nomogram") ) in All Text | 55 |

**Table S2. Distribution of lymph node–positive and lymph node–negative cases across training, validation, and external test cohorts in the included studies**

| **ID** | **Training cohort** | | **Validation cohort** | | **External test cohorts** | |
| --- | --- | --- | --- | --- | --- | --- |
|  | **LN +** | **LN-** | **LN +** | **LN-** | **LN+** | **LN-** |
| Zhong, 2018 | 373 | 70 | 41 | 8 | − | − |
| Das, 2021 | 39 | 74 | 17 | 33 | 31 | 22 |
| Zhang, 2021 | 34 | 126 | − | − | 21 | 63 |
| Liu, 2024 | 91 | 91 | 38 | 38 | − | − |
| Tian, 2024 | 235 | 235 | 101 | 101 | 142 | 511 |
| Ye, 2024 | 48 | 356 | − | − | 8 | 61 |
| Huang, 2025a | 121 | 640 | 38 | 289 | 6 | 38 |
| Huang, 2025b | 117 | 622 | 42 | 276 | 6 | 36 |
| Yin, 2025 | 93 | 93 | 24 | 24 | 42 | 82 |
| Zhao, 2025 | 59 | 436 | 18 | 106 | 20 | 113 |

**Table S3. Radiomic quality scores for all included studies**

| **ID** | **Image protocol quality** | **Multiple segmentationsy** | **Phantom study** | **Imaging at multiple time points** | **Feature reduction** | **Multivariable analysis** | **Validation** | **Discrimination + Calibration** | **Compare to “standard”** | **Potential clinical utility** | **Discuss biological correlates** | **Cost-effectiveness analyses** | **Open science and data** | **Cut-off analyses** | **Prospective study registered in a trial database** | **Multi-center study** | **Total scores** |
| --- | --- | --- | --- | --- | --- | --- | --- | --- | --- | --- | --- | --- | --- | --- | --- | --- | --- |
| Zhong, 2018 | 2 | 1 | 0 | 0 | 3 | 1 | 2 | 1 | 1 | 0 | 0 | 0 | 0 | 1 | 0 | 0 | 12 |
| Das, 2021 | 2 | 1 | 0 | 0 | 3 | 1 | 4 | 2 | 2 | 2 | 0 | 0 | 0 | 1 | 0 | 3 | 21 |
| Zhang, 2021 | 2 | 1 | 0 | 0 | 3 | 1 | 4 | 2 | 1 | 2 | 0 | 0 | 0 | 1 | 0 | 3 | 20 |
| Liu 2024 | 2 | 1 | 0 | 0 | 3 | 1 | 2 | 2 | 2 | 2 | 0 | 0 | 0 | 0 | 0 | 0 | 15 |
| Tian, 2024 | 2 | 1 | 0 | 0 | 3 | 0 | 4 | 1 | 1 | 0 | 0 | 0 | 2 | 0 | 0 | 3 | 17 |
| Ye, 2024 | 2 | 1 | 0 | 0 | 3 | 1 | 4 | 2 | 2 | 2 | 1 | 0 | 0 | 1 | 0 | 3 | 22 |
| Huang, 2025a | 2 | 0 | 0 | 0 | 3 | 1 | 4 | 2 | 2 | 2 | 0 | 0 | 0 | 0 | 0 | 3 | 19 |
| Huang, 2025b | 2 | 0 | 0 | 0 | 3 | 1 | 4 | 2 | 2 | 2 | 0 | 0 | 0 | 1 | 0 | 3 | 20 |
| Yin, 2025 | 2 | 0 | 0 | 0 | 3 | 1 | 4 | 1 | 2 | 2 | 0 | 0 | 0 | 0 | 0 | 3 | 18 |
| Zhao, 2025 | 2 | 1 | 0 | 0 | 3 | 1 | 4 | 2 | 2 | 2 | 0 | 0 | 0 | 1 | 0 | 3 | 21 |

**Figure S1. Forest plots of PLR and NLR with corresponding heterogeneity statistics. A, internal validation cohorts; B, external validation.**


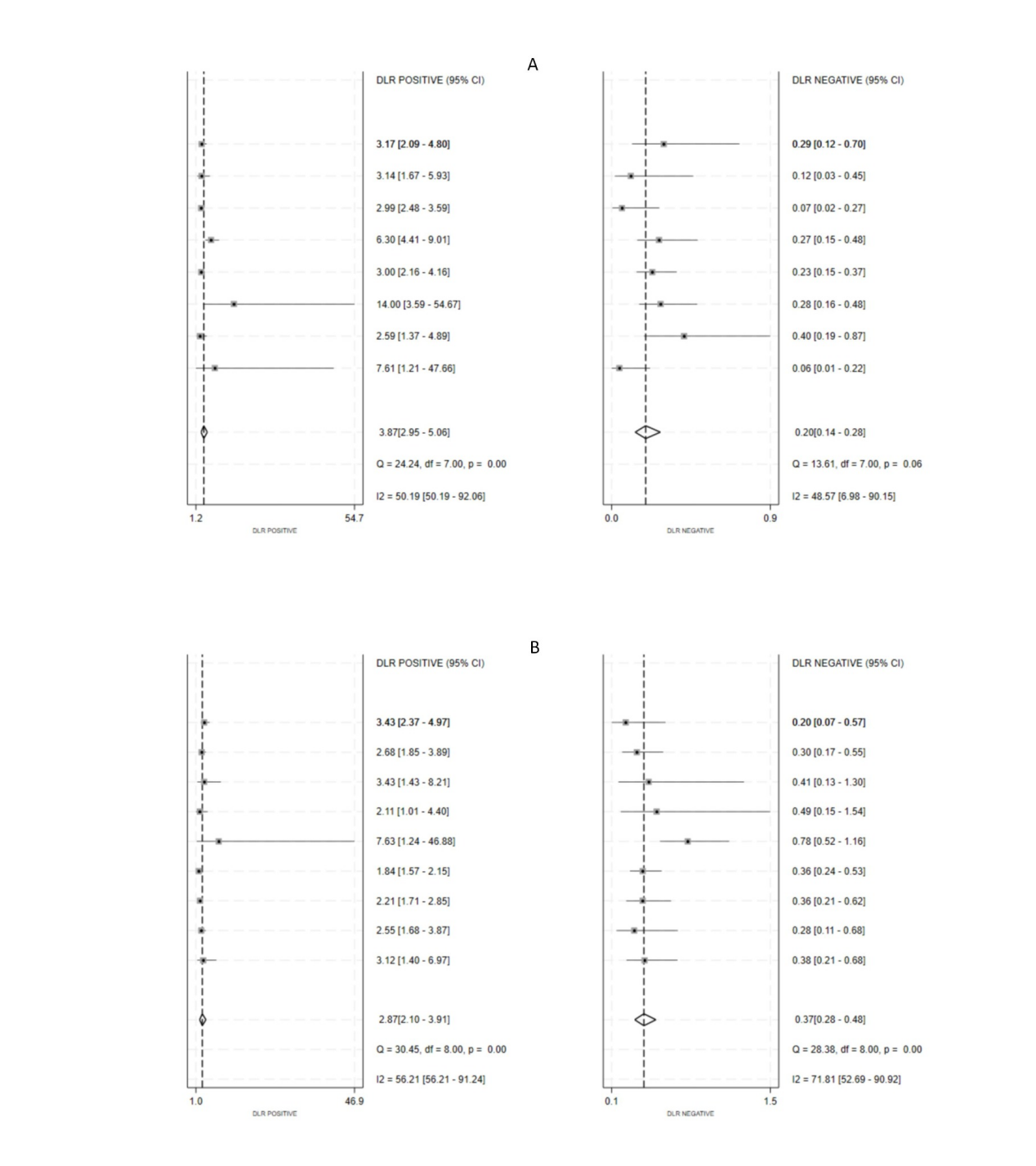


**Figure S2. Forest plots of DOR with corresponding heterogeneity statistics. A, internal validation cohorts; B, external validation.**

**
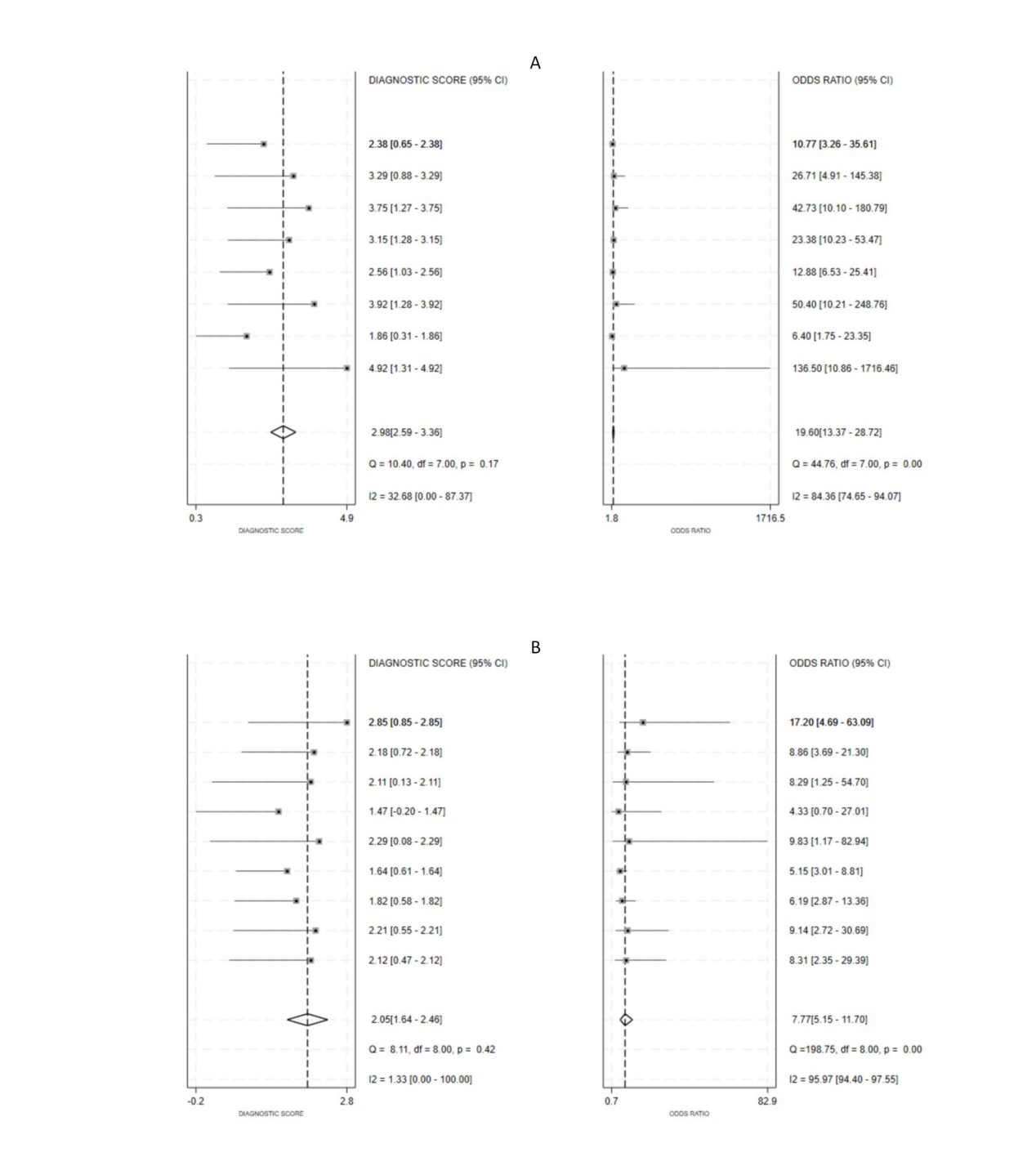
**

**Figure S3. Fagan nomogram of CT-based radiomics for diagnosis of occult lymph node metastasis according to different pretest probabilities (prior). A, internal validation cohorts; B, external validation.**

**
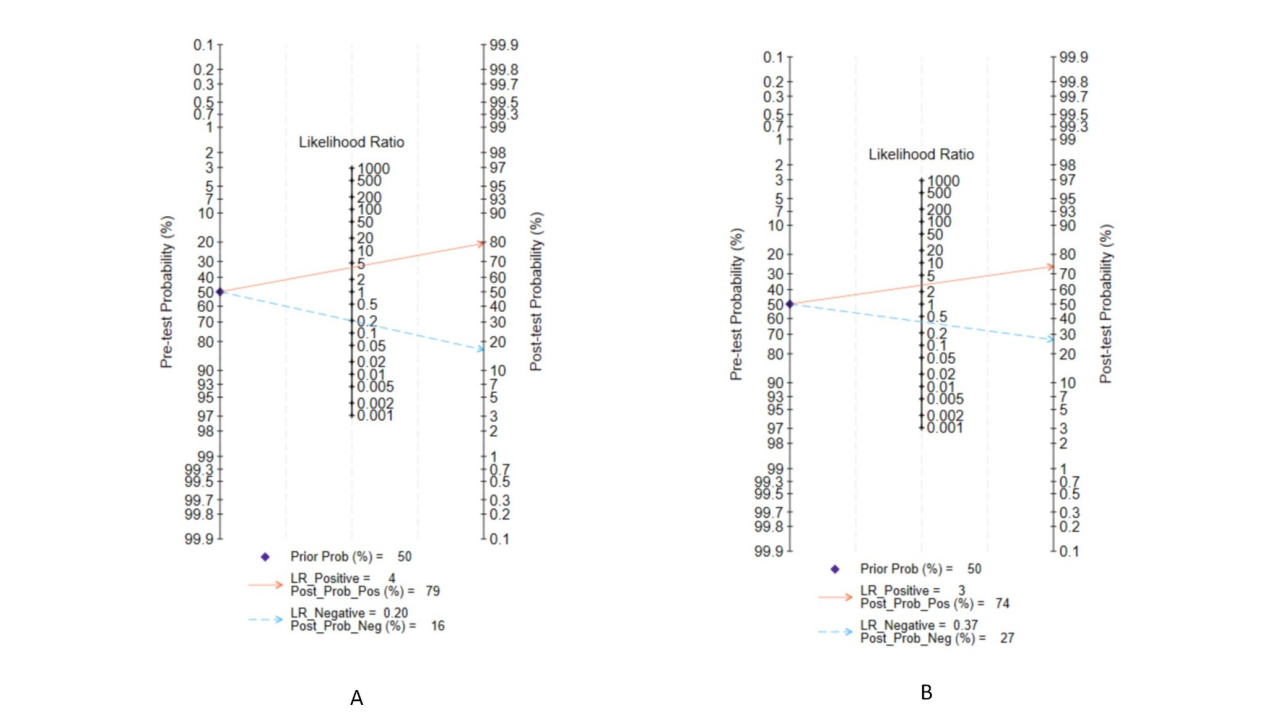
**

**Figure S4. Negative Likelihood ratio scattergram. A, internal validation cohorts; B, external validation.**

**
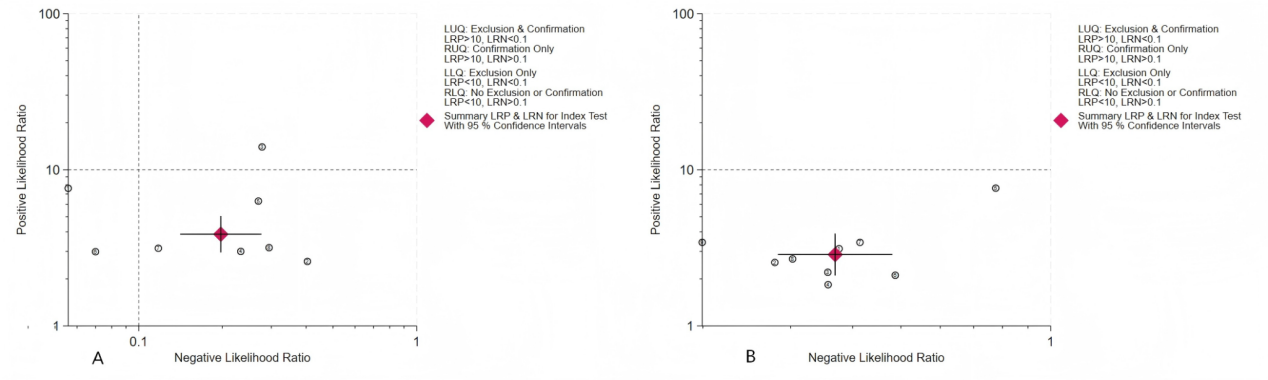
**
